# 1-{5-[2-Chloro-5-(trifluoro­meth­yl)phen­yl]thio­phen-2-yl}ethanone

**DOI:** 10.1107/S1600536811003965

**Published:** 2011-02-09

**Authors:** Roman Lytvyn, Yuri Horak, Vasyl Matiychuk, Mykola Obushak, Vasyl Kinzhybalo

**Affiliations:** a Department of Organic Chemistry, Ivan Franko National University of Lviv, Kyryla and Mefodiya 6, Lviv 79005, Ukraine; b Faculty of Chemistry, University of Wrocław, 14 Joliot-Curie St, 50-383 Wrocław, Poland

## Abstract

In the title molecule, C_13_H_8_ClF_3_OS, the dihedral angle between the mean planes of 2-chloro-5-(trifluoro­meth­yl)phenyl and tiophene rings is 54.37 (5)°. The acethyl group is twisted by 8.1 (2)° with respect to the thio­phene ring. The CF_3_ group is disordered over two sets of sites with occupations of 0.49 (3) and 0.51 (3). The crystal packing features C—H⋯F and C—H⋯O hydrogen bonds, forming dimers which are connected into chains along the *c* axis by C—H⋯O hydrogen bonds and C—Cl⋯π [Cl⋯π = 3.415 (1) Å and C—Cl⋯π = 151.56 (5)°] inter­actions. The chains are further connected into layers perpendicular to the *a* axis by C—H⋯O inter­actions.

## Related literature

For the general synthetic procedure, see: Matiychuk *et al.* (2010 ▶). For the biologial activity of aryl­thio­phenes, see: Reddy *et al.* (2005 ▶); Anderson *et al.* (1963 ▶); Bohlmann *et al.* (1984 ▶); Michaelides *et al.* (1997 ▶); Tanaka *et al.* (1998 ▶) and for their applications, see Masui *et al.* (2004 ▶); Roncali (1992 ▶, 1997 ▶). For methods of obtaining aryl­thio­phenes *via* cross-coupling reactions, see: Stanforth (1998 ▶). 
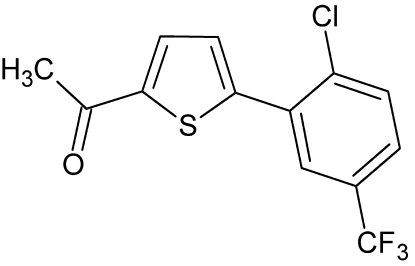


## Experimental

### 

#### Crystal data

C_13_H_8_ClF_3_OS
*M* *_r_* = 304.70
Monoclinic, 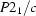

*a* = 15.330 (6) Å
*b* = 10.809 (4) Å
*c* = 7.676 (3) Å
β = 93.72 (3)°
*V* = 1269.3 (8) Å^3^
*Z* = 4
Mo *K*α radiation
μ = 0.49 mm^−1^
*T* = 100 K
0.20 × 0.15 × 0.08 mm

#### Data collection

Kuma KM-4-CCD diffractometer
Absorption correction: analytical (*CrysAlis RED*; Oxford Diffraction, 2006 ▶) *T*_min_ = 0.86, *T*_max_ = 0.93
16021 measured reflections
4377 independent reflections
3093 reflections with *I* > 2σ(*I*)
*R*_int_ = 0.034

#### Refinement

*R*[*F* ^2^ > 2σ(*F* ^2^)] = 0.039
*wR*(*F* ^2^) = 0.097
*S* = 1.00
4377 reflections
201 parameters
H-atom parameters constrained
Δρ_max_ = 0.49 e Å^−3^
Δρ_min_ = −0.25 e Å^−3^

### 

Data collection: *CrysAlis CCD* (Oxford Diffraction, 2006 ▶); cell refinement: *CrysAlis RED* (Oxford Diffraction, 2006 ▶); data reduction: *CrysAlis RED*; program(s) used to solve structure: *SHELXS97* (Sheldrick, 2008 ▶); program(s) used to refine structure: *SHELXL97* (Sheldrick, 2008 ▶); molecular graphics: *DIAMOND* (Brandenburg, 2006) ▶; software used to prepare material for publication: *publCIF* (Westrip, 2010 ▶).

## Supplementary Material

Crystal structure: contains datablocks I, global. DOI: 10.1107/S1600536811003965/ds2088sup1.cif

Structure factors: contains datablocks I. DOI: 10.1107/S1600536811003965/ds2088Isup2.hkl

Additional supplementary materials:  crystallographic information; 3D view; checkCIF report

## Figures and Tables

**Table 1:** Hydrogen-bond geometry (Å, °)

*D*—H⋯*A*	*D*—H	H⋯*A*	*D*⋯*A*	*D*—H⋯*A*
C22—H22*C*⋯F1*A*^i^	0.98	2.55	3.520 (13)	168
C22—H22*B*⋯O1^ii^	0.98	2.62	3.526 (2)	154
C22—H22*A*⋯O1^iii^	0.98	2.67	3.562 (3)	152
C56—H56⋯O1^i^	0.95	2.78	3.697 (2)	162

Symmetry codes: (i) 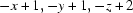; (ii) 
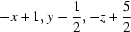
; (iii) 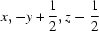.

**Fig. 1.**
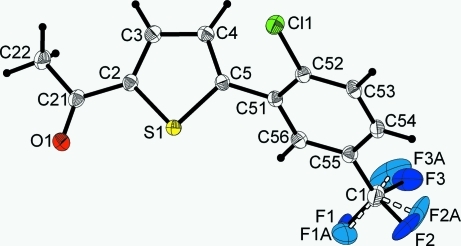
Asymmetric unit of the crystal alnog with atom labeling scheme. The thermal ellipsoids are drawn at the 50% probability level.

**Fig. 2.**
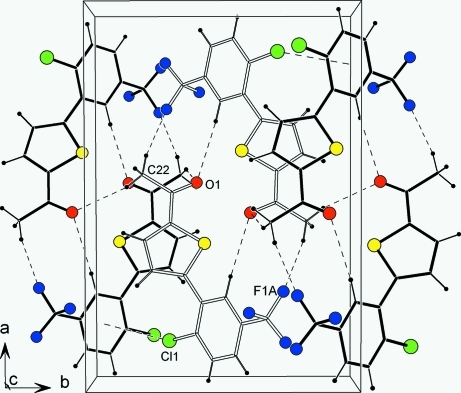
The packing of the title compound along with intermolecular hydrogen bonds.

## supplementary crystallographic information

### Comment

Arylthiophenes and their homologues are an important class of organic compounds. The arylthiophenes units are represented in several types of compounds of current interest including polymers (Roncali, 1992; Roncali, 1997), liquid crystals (Masui *et al.*, 2004), ligands and molecules of medicinal interest (Michaelides *et al.*, 1997; Tanaka *et al.*, 1998; Reddy *et al.*, 2005; Anderson *et al.*, 1963). In view of the arylthiophenes importance a number of catalytic methods of these compounds formation from precursors in a cross-coupling reactions have been developed over the last two decades (Stanforth, 1998). However, these methods proceed in two steps *via* an organometallic intermediate and their stability is often limited.

The molecule of the title compound is not plannar (see Fig. 1). The dihedral angle between the mean planes of 2-chloro-5-(trifluoromethyl)phenyl and tiophene rings is equal to 54.37 (5)°. The acethyl group is twisted with respect to the tiophene ring by 8.1 (2)°. The CF_3_ group is disordered with almoust equal occupations of two positions, which are realised by the rotation around C1–C55 bond. The crystal structure packing is governed by the hydrogen bonds of C–H···F and C–H···O types and C–Cl···π interactions. The centrosymmetric dimers are formed by the pairs of C22–H22C···F1A*^i^* and C56–H56···O1*^i^* hydrogen bonds. The dimers are connected into the chains that propagate along *z* axis direction by means of C22–H22A···O1*^iii^* hydrogen bonds and C52–Cl1···*C_g_^iv^* interactions (symmetry code: (*iv*) *x*, 1/2 - *y*, *z* + 1/2). The geometrical parameters of C52–Cl1···*C_g_^iv^* interaction are as follows: Cl1···*C_g_^iv^* distance is equal to 3.415 (1) Å and the C52–Cl1···*C_g_^iv^* angle 151.56 (5)°. The vast layers perpendicular to *x* axis direction are formed of above mentioned chains connected with each other by C22–H22B···O1*^ii^* hydrogen bonds (see Fig. 2).

### Experimental

Water solution of 7 g of NaNO_2_ (25 ml) was added dropwise to a cooled stirred mixture of 2-chloro-5-trifluoromethylaniline (19.5 g, 0.1 mol, Fluka) and 60 ml of 20% HCl. After completion of reaction the solution was filtered and added dropwise to well stirred mixture of 2-acetylthiophene (12.6 g, 0.1 mol, Fluka), acetone (40 ml) and CuCl_2_^.^2H_2_O (1.5 g, 8.7 mmol) during 20 min. After 3 h the reaction mixture was diluted with 250 ml of water and 50 ml of CHCl_3_, organic layer was separated and dried over Na_2_SO_4_, and concentrated under reduced pressure. Residue was distilled at 400 Pa (453–458 K) and gave 11.6 g (38% yield) of 1-{5-[2-chloro-5-(trifluoromethyl)phenyl]-2-thienyl}ethanone. Yellow crystals suitable for X-ray analysis were obtained by recrystallization from *n*-hexane.

### Refinement

All H atoms were found in difference-Fourier maps. In the final refinement cycles, all H atoms were positioned geometrically and treated as riding atoms, with C–H distance of 0.95 Å and with *U*_iso_(H) values of 1.2*U*_eq_(C).

### Crystal data

**Table:**

C_13_H_8_ClF_3_OS	*F*(000) = 616
*M**_r_* = 304.70	*D*_x_ = 1.594 Mg m^−^^3^
Monoclinic, *P*2_1_/*c*	Melting point: 347 K
Hall symbol: -P 2ybc	Mo *K*α radiation, λ = 0.71073 Å
*a* = 15.330 (6) Å	Cell parameters from 11592 reflections
*b* = 10.809 (4) Å	θ = 2.3–33.9°
*c* = 7.676 (3) Å	µ = 0.49 mm^−^^1^
β = 93.72 (3)°	*T* = 100 K
*V* = 1269.3 (8) Å^3^	Block, colourless
*Z* = 4	0.20 × 0.15 × 0.08 mm

### Data collection

**Table:**

Kuma KM-4-CCD diffractometer	4377 independent reflections
Radiation source: fine-focus sealed tube	3093 reflections with *I* > 2σ(*I*)
graphite	*R*_int_ = 0.034
ω scans	θ_max_ = 33.8°, θ_min_ = 3.3°
Absorption correction: analytical (*CrysAlis RED*; Oxford Diffraction, 2006)	*h* = −19→23
*T*_min_ = 0.86, *T*_max_ = 0.93	*k* = −12→16
16021 measured reflections	*l* = −11→9

### Refinement

**Table:**

Refinement on *F*^2^	Primary atom site location: structure-invariant direct methods
Least-squares matrix: full	Secondary atom site location: difference Fourier map
*R*[*F*^2^ > 2σ(*F*^2^)] = 0.039	Hydrogen site location: inferred from neighbouring sites
*wR*(*F*^2^) = 0.097	H-atom parameters constrained
*S* = 1.00	*w* = 1/[σ^2^(*F*_o_^2^) + (0.054*P*)^2^] where *P* = (*F*_o_^2^ + 2*F*_c_^2^)/3
4377 reflections	(Δ/σ)_max_ = 0.001
201 parameters	Δρ_max_ = 0.49 e Å^−^^3^
0 restraints	Δρ_min_ = −0.25 e Å^−^^3^

### Special details

**Table:**

Geometry. All e.s.d.'s (except the e.s.d. in the dihedral angle between two l.s. planes) are estimated using the full covariance matrix. The cell e.s.d.'s are taken into account individually in the estimation of e.s.d.'s in distances, angles and torsion angles; correlations between e.s.d.'s in cell parameters are only used when they are defined by crystal symmetry. An approximate (isotropic) treatment of cell e.s.d.'s is used for estimating e.s.d.'s involving l.s. planes.
Refinement. Refinement of *F*^2^ against ALL reflections. The weighted *R*-factor *wR* and goodness of fit *S* are based on *F*^2^, conventional *R*-factors *R* are based on *F*, with *F* set to zero for negative *F*^2^. The threshold expression of *F*^2^ > σ(*F*^2^) is used only for calculating *R*-factors(gt) *etc*. and is not relevant to the choice of reflections for refinement. *R*-factors based on *F*^2^ are statistically about twice as large as those based on *F*, and *R*- factors based on ALL data will be even larger.

### Fractional atomic coordinates and isotropic or equivalent isotropic displacement parameters (Å^2^)

**Table:**

	*x*	*y*	*z*	*U*_iso_*/*U*_eq_	Occ. (<1)
Cl1	0.09807 (2)	0.28619 (3)	1.05277 (5)	0.02440 (10)	
S1	0.37102 (2)	0.42596 (3)	1.04508 (5)	0.01971 (9)	
O1	0.54239 (7)	0.39388 (9)	1.24469 (14)	0.0261 (2)	
C2	0.42729 (9)	0.29067 (12)	1.08963 (19)	0.0181 (3)	
C21	0.51398 (9)	0.29623 (12)	1.18365 (19)	0.0208 (3)	
C22	0.56531 (10)	0.17781 (14)	1.1983 (2)	0.0276 (3)	
H22A	0.5812	0.1521	1.0821	0.041*	
H22B	0.5297	0.1133	1.2485	0.041*	
H22C	0.6185	0.1909	1.2738	0.041*	
C3	0.37969 (9)	0.18899 (12)	1.02942 (19)	0.0194 (3)	
H3	0.4000	0.1062	1.0413	0.023*	
C4	0.29753 (9)	0.22147 (12)	0.94833 (19)	0.0192 (3)	
H4	0.2562	0.1630	0.9010	0.023*	
C5	0.28396 (9)	0.34750 (11)	0.94559 (18)	0.0160 (3)	
C51	0.20972 (9)	0.41760 (11)	0.86157 (18)	0.0164 (3)	
C52	0.12277 (9)	0.39514 (12)	0.89694 (18)	0.0179 (3)	
C53	0.05343 (9)	0.45875 (12)	0.81028 (19)	0.0192 (3)	
H53	−0.0051	0.4413	0.8357	0.023*	
C54	0.07070 (9)	0.54733 (12)	0.68726 (19)	0.0198 (3)	
H54	0.0241	0.5907	0.6270	0.024*	
C55	0.15699 (9)	0.57247 (12)	0.65236 (18)	0.0192 (3)	
C1	0.17567 (10)	0.66394 (15)	0.5124 (2)	0.0289 (4)	
F1	0.2540 (6)	0.7124 (13)	0.528 (2)	0.049 (3)	0.49 (3)
F2	0.1209 (7)	0.7645 (7)	0.5195 (13)	0.0485 (17)	0.49 (3)
F3	0.1634 (9)	0.6244 (10)	0.3552 (8)	0.059 (2)	0.49 (3)
F1A	0.2471 (7)	0.7292 (12)	0.549 (2)	0.052 (2)	0.51 (3)
F2A	0.1125 (5)	0.7352 (16)	0.465 (2)	0.070 (3)	0.51 (3)
F3A	0.1942 (11)	0.5975 (8)	0.3643 (11)	0.079 (2)	0.51 (3)
C56	0.22588 (9)	0.50955 (12)	0.73913 (18)	0.0189 (3)	
H56	0.2843	0.5290	0.7153	0.023*	

### Atomic displacement parameters (Å^2^)

**Table:**

	*U*^11^	*U*^22^	*U*^33^	*U*^12^	*U*^13^	*U*^23^
Cl1	0.01931 (18)	0.02759 (17)	0.0267 (2)	−0.00030 (13)	0.00436 (14)	0.00743 (14)
S1	0.01739 (17)	0.01659 (14)	0.0246 (2)	−0.00050 (12)	−0.00288 (13)	0.00172 (13)
O1	0.0217 (5)	0.0253 (5)	0.0307 (6)	−0.0040 (4)	−0.0041 (5)	0.0034 (4)
C2	0.0151 (6)	0.0200 (6)	0.0193 (7)	0.0012 (5)	0.0017 (5)	0.0023 (5)
C21	0.0178 (7)	0.0234 (6)	0.0214 (8)	−0.0002 (5)	0.0024 (6)	0.0072 (5)
C22	0.0194 (7)	0.0266 (7)	0.0362 (10)	0.0026 (6)	−0.0034 (7)	0.0065 (6)
C3	0.0184 (7)	0.0194 (6)	0.0208 (7)	0.0027 (5)	0.0033 (6)	0.0005 (5)
C4	0.0201 (7)	0.0185 (6)	0.0189 (7)	−0.0012 (5)	0.0009 (6)	−0.0014 (5)
C5	0.0154 (6)	0.0177 (5)	0.0149 (7)	−0.0002 (5)	0.0009 (5)	−0.0005 (5)
C51	0.0164 (6)	0.0170 (5)	0.0158 (7)	0.0011 (5)	0.0002 (5)	−0.0031 (5)
C52	0.0185 (7)	0.0190 (5)	0.0163 (7)	0.0000 (5)	0.0009 (5)	−0.0009 (5)
C53	0.0138 (6)	0.0234 (6)	0.0203 (7)	0.0012 (5)	0.0007 (5)	−0.0041 (5)
C54	0.0182 (7)	0.0217 (6)	0.0190 (7)	0.0028 (5)	−0.0027 (6)	−0.0021 (5)
C55	0.0200 (7)	0.0200 (6)	0.0174 (7)	0.0005 (5)	0.0005 (5)	0.0003 (5)
C1	0.0219 (8)	0.0343 (8)	0.0300 (9)	0.0007 (6)	−0.0013 (7)	0.0102 (7)
F1	0.015 (2)	0.068 (5)	0.062 (5)	0.000 (3)	−0.003 (2)	0.043 (4)
F2	0.050 (3)	0.038 (2)	0.059 (4)	0.0255 (18)	0.020 (2)	0.0291 (19)
F3	0.115 (6)	0.043 (3)	0.0173 (15)	−0.024 (3)	−0.005 (2)	0.0037 (17)
F1A	0.053 (5)	0.049 (3)	0.053 (3)	−0.029 (3)	−0.008 (3)	0.022 (2)
F2A	0.0206 (16)	0.093 (6)	0.099 (7)	0.016 (3)	0.010 (3)	0.074 (5)
F3A	0.160 (6)	0.051 (2)	0.029 (2)	0.010 (4)	0.037 (3)	0.0148 (18)
C56	0.0169 (7)	0.0211 (6)	0.0189 (7)	−0.0004 (5)	0.0018 (5)	−0.0017 (5)

### Geometric parameters (Å, °)

**Table:**

Cl1—C52	1.738 (2)	C51—C56	1.401 (2)
S1—C5	1.718 (2)	C52—C53	1.398 (2)
S1—C2	1.721 (2)	C53—C54	1.382 (2)
O1—C21	1.224 (2)	C53—H53	0.9500
C2—C3	1.382 (2)	C54—C55	1.393 (2)
C2—C21	1.472 (2)	C54—H54	0.9500
C21—C22	1.503 (2)	C55—C56	1.389 (2)
C22—H22A	0.9800	C55—C1	1.501 (2)
C22—H22B	0.9800	C1—F2A	1.272 (8)
C22—H22C	0.9800	C1—F3	1.282 (8)
C3—C4	1.413 (2)	C1—F1	1.309 (10)
C3—H3	0.9500	C1—F1A	1.317 (11)
C4—C5	1.378 (2)	C1—F2	1.377 (7)
C4—H4	0.9500	C1—F3A	1.389 (8)
C5—C51	1.480 (2)	C56—H56	0.9500
C51—C52	1.399 (2)		
			
C5—S1—C2	91.94 (7)	C53—C52—Cl1	117.91 (11)
C3—C2—C21	129.54 (12)	C51—C52—Cl1	120.28 (11)
C3—C2—S1	111.25 (11)	C54—C53—C52	119.50 (13)
C21—C2—S1	119.20 (10)	C54—C53—H53	120.3
O1—C21—C2	120.70 (13)	C52—C53—H53	120.3
O1—C21—C22	122.31 (14)	C53—C54—C55	119.52 (13)
C2—C21—C22	116.98 (12)	C53—C54—H54	120.2
C21—C22—H22A	109.5	C55—C54—H54	120.2
C21—C22—H22B	109.5	C56—C55—C54	120.92 (13)
H22A—C22—H22B	109.5	C56—C55—C1	119.42 (13)
C21—C22—H22C	109.5	C54—C55—C1	119.58 (13)
H22A—C22—H22C	109.5	F3—C1—F1	107.4 (8)
H22B—C22—H22C	109.5	F2A—C1—F1A	110.1 (7)
C2—C3—C4	112.70 (12)	F3—C1—F2	104.4 (6)
C2—C3—H3	123.7	F1—C1—F2	103.8 (7)
C4—C3—H3	123.7	F2A—C1—F3A	105.6 (6)
C5—C4—C3	112.45 (12)	F1A—C1—F3A	103.8 (8)
C5—C4—H4	123.8	F2A—C1—C55	115.3 (4)
C3—C4—H4	123.8	F3—C1—C55	115.5 (4)
C4—C5—C51	128.60 (12)	F1—C1—C55	114.4 (6)
C4—C5—S1	111.66 (10)	F1A—C1—C55	113.4 (6)
C51—C5—S1	119.62 (10)	F2—C1—C55	110.3 (4)
C52—C51—C56	117.75 (12)	F3A—C1—C55	107.7 (4)
C52—C51—C5	122.83 (12)	C55—C56—C51	120.46 (13)
C56—C51—C5	119.41 (12)	C55—C56—H56	119.8
C53—C52—C51	121.81 (13)	C51—C56—H56	119.8

### Hydrogen-bond geometry (Å, °)

**Table:**

*D*—H···*A*	*D*—H	H···*A*	*D*···*A*	*D*—H···*A*
C22—H22C···F1A^i^	0.98	2.55	3.520 (13)	168
C22—H22B···O1^ii^	0.98	2.62	3.526 (2)	154
C22—H22A···O1^iii^	0.98	2.67	3.562 (3)	152
C56—H56···O1^i^	0.95	2.78	3.697 (2)	162

Symmetry codes: (i) −*x*+1, −*y*+1, −*z*+2; (ii) −*x*+1, *y*−1/2, −*z*+5/2; (iii) *x*, −*y*+1/2, *z*−1/2.
